# Lived Experiences of Mothers With Preterm Infants Providing Kangaroo Mother Care

**DOI:** 10.1002/nop2.70760

**Published:** 2026-08-13

**Authors:** Marzieh Avazeh, Shahla Shahbazi, Leili Borimnejad, Mohammadbagher Hosseini, Mahnaz Jabraeili

**Affiliations:** ^1^ Department of Pediatric Nursing, Faculty of Nursing and Midwifery Tabriz University of Medical Sciences Tabriz Iran; ^2^ Department of Medical Surgical Nursing, Faculty of Nursing and Midwifery Tabriz University of Medical Sciences Tabriz Iran; ^3^ Clinical Research Development Unit, Sina Education, Research and Treatment Center Tabriz University of Medical Sciences Tabriz Iran; ^4^ Neonatal and Pediatric Nursing Department Nursing and Midwifery School, Iran University of Medical Sciences Tehran Iran; ^5^ Neonatologist, Pediatric Health Research Centre Tabriz University of Medical Sciences Tabriz Iran

**Keywords:** experience, kangaroo mother care, mother, phenomenology, preterm, qualitative study

## Abstract

**Aim:**

The aim of this study was to explore the lived experiences of mothers with preterm infants providing kangaroo mother care.

**Design:**

A qualitative phenomenological design.

**Methods:**

In‐depth unstructured interviews were conducted over six months, from January to June 2024, with mothers of preterm infants who were providing kangaroo mother care for at least one week. The seven‐step method was used to analyse the data.

**Results:**

Ten mothers with hospitalized preterm infants participated in this study. A constitutive pattern of “life‐giving refuge” and three main themes emerged from the lived experiences of mothers with preterm infants providing kangaroo mother care: (1) development of maternal attachment, (2) gradual empowerment, and (3) gaining confidence through positive outcomes.

**Conclusions:**

The present study highlighted the benefits of kangaroo mother care in fostering emotional bonds between mothers and infants. Additionally, it emphasized the importance of proper training, continuous support, and monitoring by healthcare professionals to maximize the effectiveness of kangaroo mother care. The findings of our study can be utilized to develop educational content for nurses and physicians to ensure the optimal implementation of kangaroo mother care. This includes explaining the benefits of kangaroo mother care for both mothers and infants, identifying mothers' concerns regarding its practice, and providing effective solutions to address those concerns.

**Implications for Nursing Practice:**

Neonatal nurses can facilitate kangaroo mother care by providing individualized education, hands‐on coaching, emotional reassurance, and ongoing encouragement to help mothers build confidence and overcome fear and uncertainty.

**Reporting Method:**

This study adhered to the SRQR reporting guideline.

**Patient or Public Contribution:**

No patient or public contribution.

## 1 Introduction

Preterm birth is a major global health problem. Every year, 13.4 million preterm infants are born worldwide, meaning that one in every ten babies is born prematurely (World Health Organization 2023). In Iran, the prevalence of preterm birth has been estimated to be approximately 10%, indicating that preterm birth remains a significant public health concern (Khezri et al. 2025). Prematurity is the leading cause of neonatal mortality, and it increases the risk of respiratory distress syndrome, cerebral palsy, and developmental abnormalities. In addition, preterm infants are at increased risk of developing chronic non‐communicable diseases later in life due to interference with organ system development during the intrauterine and perinatal periods (Morniroli et al. 2023).

The birth of a preterm infant is often an unexpected and profoundly stressful experience for mothers. Mothers of preterm infants commonly experience fear, anxiety, uncertainty, and feelings of helplessness due to concerns about their infant's survival, health, and future development. Despite these emotional challenges, mothers are often highly motivated to participate in interventions that may improve their infant's health and well‐being (Mehrpisheh et al. 2023; Pathak et al. 2023). According to the Theory of Reasoned Action, individuals' attitudes and beliefs about a behaviour influence their behavioural intentions. Therefore, mothers' perceptions of the potential benefits of health‐promoting interventions may influence their intentions to engage in such behaviours (Fishbein and Ajzen 1975).

The developmental care approach, aimed at improving infant development, was designed based on the synactive theory presented by Dr. Heidelise Als (Als 1982). Developmental care is implemented to neuroprotect preterm infants from the extrauterine environment and includes various concepts and interventions, including kangaroo mother care (KMC) (Burke 2018). KMC is prolonged skin‐to‐skin contact (SSC) between the mother and infant immediately after birth and continues until the corrected gestational age reaches 40 weeks (Li et al. 2022; Nguyen et al. 2019). KMC is an approach to providing neonatal care with a long history (Jadhao et al. 2020) and has been recommended by the World Health Organization (WHO) as routine care for all preterm or low‐birth‐weight infants (World Health Organization 2022).

KMC is a simple, effective, and cost‐effective method with sufficient evidence confirming its many benefits for infants and parents (Bharadwaj and Iqbal 2024; Jadhao et al. 2020; Nguyen et al. 2019). A study conducted on 600 preterm infants reported that KMC can positively impact neonatal outcomes, including accelerated weight gain, reduced dependence on oxygen, reduced length of hospitalization, and the rate of infant readmission (Saeidi hasani and Valizadeh 2021). Other studies on KMC have reported benefits, including helping to improve the infant's vital signs (breathing, oxygenation, heart rate, and temperature), deep sleep, analgesic effects, exclusive breastfeeding for up to 6 months (Zirpoli et al. 2019), prevention of hospital‐acquired infections (Jadhao et al. 2020), stronger emotional bonding between mother and infant, better infant growth and development (Koreti and Gharde 2022), and reduced mortality (Nguyen et al. 2019). Starting KMC immediately after birth and performing it regularly has long‐term benefits, including reduced susceptibility to illness in low‐birth‐weight and preterm infants (Bharadwaj and Iqbal 2024) and improved neurodevelopmental outcomes regarding cognition, language, and adaptive behaviour at 12 months of age (Bisanalli et al. 2023). In addition to benefiting the infant, KMC also positively affects maternal health. These benefits include reducing maternal stress and postpartum depression (Cristóbal Cañadas et al. 2022; Herizchi et al. 2017), improving sleep quality (Chen et al. 2022), and increasing resilience in mothers with preterm infants (Abasalizadeh et al. 2024).

Despite the well‐known benefits of KMC, this intervention has not yet been fully integrated into healthcare systems worldwide (Salim et al. 2021; Stefani et al. 2022). Effective integration of KMC into existing health systems will significantly improve infant survival rates by removing barriers and building trust. KMC implementation issues are likely to vary across regions. Therefore, there is a need for further research into sustainable development mechanisms to promote KMC adoption in different settings (Cai et al. 2022).

Although KMC is a relatively simple intervention, its consistent implementation requires support from the health system, staff, mothers, families, and communities (Rahmatika et al. 2022). Social support has been recognized as an effective factor in reducing parenting stress (Ghanizadeh et al. 2023). Parents of preterm infants and other family members, the medical unit, and the medical staff constitute a closely interconnected and dynamic triangle. In order to successfully integrate KMC into daily care, the support of health center managers, training of medical staff, and education and encouragement of parents of preterm infants and other family members are essential (Cai et al. 2022). Meanwhile, healthcare providers and mothers are the leading actors in implementing KMC, and establishing a good interpersonal relationship between healthcare providers and mothers will be very beneficial (Rahmatika et al. 2022).

Several studies have examined the barriers to implementing KMC and the factors that facilitate it. Some of these studies have pointed to differences in perceived barriers due to cultural differences and existing infrastructure and facilities (Mohammadi et al. 2021). A recent qualitative study examining caregivers' experiences with KMC showed that certain social and cultural conditions can facilitate KMC implementation (Sjömar et al. 2023). A study that reviewed the experiences of mothers with preterm infants regarding KMC in sub‐Saharan Africa reported that further research is needed to gain a deeper understanding of the varying acceptance of KMC by mothers and the behaviours, assumptions, and cultures that may influence the practice (Bayo et al. 2022). On the other hand, among the qualitative studies conducted in this field, less attention has been given to mothers' lived experiences of KMC in different settings using phenomenology (Ndou et al. 2021). Even though the center under study is the NIDCAP hub in Iran and more than 1000 preterm infants are admitted there each month, the mothers' experiences of KMC have not been studied so far. According to previous studies, experiences are context‐dependent, and these experiences may differ across cultures. Therefore, identifying and deeply understanding mothers' experiences of providing KMC can help improve its implementation. To achieve this, the researchers used an interpretive phenomenological approach. The aim of this study was to explore the lived experiences of mothers with preterm infants providing kangaroo mother care.

## 2 Methods

### 2.1 Design

This qualitative study used a hermeneutic interpretive phenomenological approach based on Heideggerian philosophy to explore the lived experiences of mothers with preterm infants providing KMC. This qualitative research method is used when the research question seeks the meanings of a phenomenon to understand human experience (Crist and Tanner 2003). The purpose of interpretive phenomenology is not only to describe experience but also to interpret the hidden meanings, contexts, and underlying structures while understanding that the researcher's preconceptions are part of the interpretive process (Matua and Van Der Wal 2015; Oluka 2025). An interpretive phenomenological approach was adopted because the aim of this study was not merely to describe mothers' experiences but to interpret the meanings of their lived experiences while providing KMC within their clinical and sociocultural contexts. This approach acknowledges the researcher's pre‐understanding as an integral component of the interpretive process, which was considered appropriate given the researcher's clinical background in neonatal care.

### 2.2 Setting and Participants

The present study was conducted in the neonatal department of the largest referral center for high‐risk pregnancies at risk of preterm birth in northwest Iran. This center provides care for preterm and high‐risk infants with advanced equipment, experienced and trained physicians and nurses, and the implementation of the neonatal developmental care program. The neonatal department of the center consists of four wards: Neonatal Intensive Care Unit (NICU) 1, NICU 2, neonatal ward, and continuous KMC ward. NICUs 1 and 2 each have 25 active beds, and the neonatal ward has 30 active beds. The continuous KMC unit, which was set up 2 years ago next to the hospital's NICU, is managed under the supervision of the neonatal ward and consists of three rooms, two with four beds and one with two beds. Infants admitted to the NICU are transferred to this unit with their mothers after their respiratory status has stabilized. Mothers stay in this unit full‐time, holding their infants on their chests with skin‐to‐skin contact. There are no restrictions on the presence of mothers in this department. Mothers are always present in the hospital and can be with their infants 24 h a day. A designated area is available for mothers to stay in the ward, and amenities include a shared lounge equipped with reclining chairs and a shared bathroom and toilet for mothers. At this center, KMC begins immediately after birth, and infants are placed in their mother's embrace. After hospitalization, with the mother's first presence in the unit, KMC begins under the supervision of a nurse. KMC is performed daily by mothers in the NICU. In the continuous KMC unit, mothers perform continuous KMC throughout the morning, evening, and night shifts.

In the present study, participants were selected using purposive sampling. The sequential and emergence‐driven sampling strategy was used. In this study, sampling continued until data richness was achieved, when sampling did not provide additional information and only replicated previously collected data (Patton 2014). Mothers of preterm infants admitted to the neonatal department who had experience in providing KMC for at least 1 week and were interested in sharing their experiences and discussing the topic were invited to participate in the study. Mothers with a history of preterm birth, NICU admission, and known illness that, based on medical record or self‐report, would affect continuous presence with their infant and regular KMC were excluded from the study.

### 2.3 Data Collection

Data were collected over 6 months, from January to June 2024, through unstructured, in‐depth interviews. The interviews were conducted by the first author under the supervision of a senior author. The executive researcher responsible for data collection has 7 years of experience working in paediatric and neonatal wards as a clinical nurse, has trained nursing students as an instructor in the neonatal ward for 5 years, and has research experience involving preterm infants during their master's thesis. Eleven interviews were conducted with ten participants. The interviewer used field notes (Crist and Tanner 2003) during the interviews and noted the mother's nonverbal communication and mental state during the interviews. Before conducting the interview, the participants were informed that the study aimed to assess their perceptions, feelings, and experiences regarding kangaroo mother care. The interviews were individual and face‐to‐face, and after coordination between the participant and the researcher regarding the interview time during the day and the interview location, all interviews were conducted in the infant admission room after providing a private environment by drawing a screen. Participants were assured that all their conversations would be confidential and would not affect the care they received from physicians and nurses. The interviews would also be entered anonymously into the researcher's personal computer, and no one other than the research team would have access to the interviews. Written informed consent was obtained from participants. After establishing rapport and gaining the participants' trust, each interview began with a broad, open‐ended question: “Please tell me about your experiences with kangaroo mother care.” As the interview progressed, exploratory and probing questions were asked based on the participants' responses to facilitate an in‐depth exploration of their lived experiences. Examples of probing questions included: “Could you explain further?”, “When you say …, what do you mean?”, “Could you tell me more about that?”, “Could you give an example?”, and “How did you feel at that time?” At the end of the interview, the participant was asked to state if there was anything left to say. After obtaining permission from the participants, all interviews were recorded using an MP3 player and then transcribed verbatim. The mean duration of the interviews was 60 min (47 to 79 min).

### 2.4 Data Analysis

The seven‐step method of Diekelmann et al. (1989) (Diekelmann et al. 1989), which is based on Heideggerian phenomenology and has a collaborative nature (Crist and Tanner 2003), was used to analyse the data. This method was selected because it provides a clear and systematic step‐by‐step framework for the analysis of hermeneutic phenomenological data (Roberts 2009). Its collaborative approach to interpretation through discussion and consensus also aligned well with the composition of the research team (Crist and Tanner 2003). This study was part of a doctoral dissertation in nursing. The research team consisted of two nursing faculty members with a background in qualitative research, especially phenomenology, one nursing faculty member with a background in qualitative research and research in the field of neonates (first supervisor), one faculty member and neonatologist with a background in preterm infant research, and a PhD candidate in nursing with a master's degree in neonatal intensive care. Data analysis was performed simultaneously with data collection. In other words, the data analysis began with the first interview. After each interview, the transcript was reviewed several times to gain a general understanding. In the second stage, an interpretive summary was written for each interview, and attempts were made to understand and extract its hidden meanings. In the third stage, the interview transcripts were analysed. Members of the research team then studied the interviews and analyses, and corrective comments were applied to the analysis text. In the fourth stage, any discrepancies in analysis and interpretation were resolved by referring to the interview transcripts or participants. In the fifth stage, themes reflecting common meanings were identified and described by comparing the interview texts. In the sixth stage, themes were evaluated and compared to identify the constitutive patterns related to the relational themes. Finally, in the seventh stage, the draft themes and pattern, along with selected excerpts from the interview transcripts, were provided to the research team for review, and suggestions were implemented in the final draft of the findings. Data were managed using MAXQDA software (version 10.0).

### 2.5 Rigour

Guba and Lincoln's criteria, including credibility, transferability, dependability, and confirmability, were used to ensure the data's rigour and trustworthiness (Lincoln and Guba 1986).

To increase the credibility of the data, a long‐term engagement with the study subject was conducted. The first author was immersed and involved in the phenomenon under study through constant presence in the neonatal department, long‐term interaction with participants, conducting interviews, listening to the interviews repeatedly, and typing and reading them several times. The executive researcher noted their experiences, beliefs, and information regarding KMC before conducting the interviews to identify possible biases. Since this work was part of a doctoral dissertation, all procedures and stages of data analysis and interpretation were carried out under the supervision of a team of researchers skilled in phenomenology and the phenomenon under study. Participants were also provided with the printed interview transcript, interpretive summary, and codes to review the data, correct possible errors, and provide additional information if necessary. Providing complete and accurate descriptions of the study setting and participants and detailed explanations of participants' experiences increased data transferability. A clear, step‐by‐step report of the research process, including data collection, data analysis, reporting of findings, and citing participant quotes, helped increase dependability. To increase confirmability, the review procedures and decisions made were carefully documented.

### 2.6 Ethical Considerations

This study is part of a doctoral dissertation in nursing approved by the Research Ethics Committee of Tabriz University of Medical Sciences (Approval ID: IR.TBZMED.REC.1402.655, approval date: 2023‐12‐04). This study adheres to the principles of the Declaration of Helsinki. Participants received adequate information about the study's aims and methods and were assured of confidentiality of their identities. Their participation in the study was voluntary; written informed consent was obtained, and they had the right to withdraw from the study at any stage.

## 3 Findings

Ten mothers with hospitalized preterm infants participated in this study. Their ages ranged from 25 to 36 years. Some had high school diplomas, and others had university degrees. Two of the mothers had given birth to twins. There were twelve infants whose gestational age at birth ranged from 26 to 32 weeks, and their birth weight ranged from 800 to 1900 g (Table 1).

**TABLE 1:** Participant characteristics.

Participant	Age (years)	Educational level	Delivery mode	Parity	Infant' gender
1	34	Diploma	Caesarean section	Multiparous	Male
2	29	Diploma	Caesarean section	Primiparous	Female
3	36	College	Caesarean section	Primiparous	Female
4	31	College	Caesarean section	Primiparous	Female–Male
5	34	College	Normal vaginal	Primiparous	Female
6	36	Diploma	Caesarean section	Multiparous	Female–Male
7	33	College	Normal vaginal	Primiparous	Male
8	32	College	Caesarean section	Primiparous	Female
9	26	Diploma	Caesarean section	Primiparous	Male
10	25	Diploma	Caesarean section	Primiparous	Male

The data analysis revealed a constitutive pattern of “life‐giving refuge”. This overarching pattern encompassed mothers' experiences of KMC, in which the relational and developmental space of skin‐to‐skin care functioned as a refuge that fostered attachment, empowerment, and confidence. The concept of refuge for both the mother and infant does not refer solely to a protective space; rather, it denotes a relational and experiential environment in which closeness with the infant, shared caregiving experiences, and time for adjustment create opportunities for growth and empowerment, even as mothers confront their own limitations and vulnerabilities. The constitutive pattern of “life‐giving refuge” was expressed in three main themes: development of maternal attachment, gradual empowerment, and gaining confidence through positive outcomes, and nine subthemes (Table 2).

**TABLE 2:** Constitutive pattern, themes and subthemes.

Constitutive pattern	Theme	Subtheme
Life‐giving refuge	Development of maternal attachment	Gradual sense of motherhood
		Closeness
		Intimacy
	Gradual empowerment	Overcoming fear and concern
		Striving for success
		Motivational support
	Gaining confidence through positive outcomes	Growth
		Calming
		Stability

### 3.1 Development of Maternal Attachment

Mothers participating in this study stated that putting their infants to their chests during KMC made them feel like mothers. A positive relationship was formed between the mother and infant, and they gradually became dependent on each other. KMC was the main factor in attachment between mother and infant. This theme consists of three subthemes: gradual sense of motherhood, closeness, and intimacy.

A participant shared that she experienced KMC for the first time with the help of a nurse. As she held her infant for the first time, she felt a profound sense of motherhood—a realization that this infant belonged to her. Gradually, her interest in and attachment to the infant grew, motivating her to extend the duration of KMC more and more.

#### 3.1.1 Gradual Sense of Motherhood

Most mothers reported that KMC was an experience that helped accelerate and strengthen their understanding of motherhood. They stated that the first time they saw their infant in the NICU, they experienced feelings such as fear, concern, and sadness, and they did not feel a sense of love or attachment to the infant. For most mothers, holding their infant during KMC was the beginning of a sense of motherhood. By doing KMC regularly, this feeling of love within the mother was strengthened, and they experienced a sense of belonging with their infant.

I did not feel anything for my baby until I held him in my arms. When they put him in my arms, I truly understood what it was like to be a mother. (P5)

Every day I did KMC, the feeling of motherhood within me grew stronger. I kept telling myself that this baby is mine. (P4)

#### 3.1.2 Closeness

Mothers experienced KMC as an enjoyable connection with their infants, gradually increasing their willingness to perform KMC. Some mothers reported that they directly observed the positive effect of KMC on improving the mother‐infant relationship, which contributed significantly to their motivation to perform KMC regularly. In addition, mothers had pleasant experiences from the moments of performing KMC, including the mother's feeling of peace, a feeling similar to pregnancy, a feeling that all the sorrows and discomforts during pregnancy were unimportant, and the feeling of embracing their infant and having a healthy child, which turned KMC into a good memory during the difficult and stressful time of hospitalization of the preterm infant.

KMC feels good. You feel like you are back in pregnancy. The movements of his hands and feet on my chest now are exactly the same as the ones in my womb. It reminds me of the seven months he was inside me. He is kicking me again like he used to when I was pregnant. I let him stay on my chest and kick more; he seems to like it. (P6)

During KMC, our hearts grew closer. We were very comfortable. (P1)

#### 3.1.3 Intimacy

Mothers participating in the present study stated that performing KMC regularly and consistently promotes intimacy and interdependence between mother and infant. By performing KMC, the mother's love and affection for the infant increased, and in addition to increasing the desire to perform KMC, a sense of dependence on holding the infant developed in the mother. Mothers found that the emotional and loving relationship that developed between them created a mutual dependence between them and the infant, such that they became extremely used to each other and became agitated and restless in each other's absence. The nurses also noticed restlessness and crying in the infants of mothers who performed KMC regularly, at times of the day when the mother was not present with the infant, and reported it to the mothers.

After I extended my KMC duration in the NICU, my baby would get restless when I was not there… he would cry. He wanted to be embraced. (P8)

After a few days of doing KMC, sometimes I would say to myself, I just gave birth; let me stay home today and rest. I could not take it anymore; I would say, no, I should get up and go hold my baby. (P9)

With KMC, my love for the baby increased; I became attached to him and constantly asked the nurses to let me hold him in my arms. (P7)

### 3.2 Gradual Empowerment

Mothers participating in this study stated that they experienced fear and stress regarding KMC in the early days. In addition, some concerns prevented them from being constantly present with the infant and performing KMC. There were some challenges along the way. On the other hand, the mother's efforts and motivational reinforcement, along with training and encouragement from nurses and physicians, resulted in the mothers' empowerment regarding KMC. The subthemes of this theme were overcoming fear and concern, striving for success, and motivational support.

A participant shared that she initially felt apprehensive about practicing KMC because her infant was so small. However, with encouragement from the nurses, she gained the confidence to give it a try. Once she began, she experienced a deep connection, feeling as if the infant that had been inside her womb was now resting on her chest. Despite her difficult physical condition, the support from physicians and nurses motivated her to practice KMC for prolonged periods.

#### 3.2.1 Overcoming Fear and Concern

Most mothers expressed fear and inability to touch and embrace their infants in the early days, and they considered the most important reason for refusing to embrace them to be the fear of harming the infant. During the first few KMC sessions, the mothers were afraid that because they did not have the necessary skills, the infant would fall, resulting in much stress. Over time, they overcame their fear and stress by continuing to perform KMC.

The nurses would tell me, ‘Come on, let us put your baby in your arms, feel the baby.’ I would say, ‘No, I do not want to.’ I was afraid to hold the baby. I was afraid I would hurt it, like break or dislocate fingers and toes. (P6)

I really wanted to embrace him, but I was scared. I thought he would fall out of my arms. (P7)

#### 3.2.2 Striving for Success

Most mothers were with their newborns in the NICU during the first few days after delivery, when they experienced their first KMC. Most of them had undergone caesarean section or had experienced some problems during and after delivery, which prevented them from being continuously and actively present in the ward and performing KMC. Mothers reported that despite these issues, they tried to be with their infants and gradually increased the amount of time they spent in the ward.

I used to come to the baby every day, but because I felt dizzy when I got up and could not walk, I would go home after a few hours. Since I started to do KMC, I gradually increased the hours I spent with the baby. (P3)

Some mothers participating in the study mentioned that despite KMC being pleasant for both mother and infant when they tried to hold the infant in their arms for a longer period, sometimes the infant did not want to continue KMC and had to be placed back in the crib. Mothers reported that things like the infant getting hungry, getting warm from contact with the mother's body, and becoming restless prevented them from continuing KMC.

After two hours of KMC, the baby's body, which was in contact with mine, was getting warm, and I was getting bored. That is when I knew he wanted to go back to his cot. (P7)

Mothers admitted that despite initial fear and anxiety when performing KMC, with practice and persistence, they could easily hold the newborn, touch it, caress it, talk to it, or sing softly. They could perform KMC independently and acquire the necessary skills to perform it efficiently.

At first, I did KMC with the help of nurses, but then when I learned to do it myself, I could do it very easily. (P1)

#### 3.2.3 Motivational Support

Mothers attributed their ability to perform KMC to the training and motivation they received from the nurses and physicians. They stated that education about the benefits of KMC for the infant and practical training in performing KMC from the very first days of being with the newborn created the necessary awareness in mothers to want to perform KMC. However, they gained the energy needed to perform KMC regularly and continuously from nurses and physicians' motivational and encouraging words. Therefore, the staff's support and encouragement of mothers is necessary for continuing KMC.

The nurses told us so many times that if you can do it and your condition is good, come to your baby and do KMC here. It is good for your baby's growth; it improves its mood and energizes them, etc. (P5)

Every time the doctor came and I was doing KMC, he would thank me and say, ‘Mom, thank you for holding the baby in your arms. You are doing a very valuable job.’ Even though it was my own baby, this statement gave me energy. It made me stay in the ward and hold my baby longer. (P8)

### 3.3 Gaining Confidence Through Positive Outcomes

Mothers gradually perceived positive changes in their infants that they attributed to KMC. They described observing the positive effects and benefits that nurses and physicians had explained to them when they first started KMC. Therefore, mothers' desire to perform KMC increased daily, and they tried to increase the duration of KMC. Mothers expressed confidence in the benefits of KMC for their infants' growth and development. This theme included the subthemes of growth, calming, and stability.

A participant described feeling calm and relaxed during KMC, as if in a peaceful sleep, while both she and her infant rested comfortably. She also noticed a tingling sensation in her breasts and increased milk production, which reinforced her positive perception of skin‐to‐skin care. These experiences motivated her to continue KMC more frequently and for longer periods, reflecting growing confidence in its benefits.

#### 3.3.1 Growth

Most mothers reported perceiving positive changes in their infants' nutrition, weight gain, and growth that they attributed to KMC. Mothers also perceived increased breast milk production and greater willingness of their infants to breastfeed during KMC. They further perceived improvements in their infants' digestion and reported less regurgitation, vomiting, and bloating. Finally, mothers believed that longer and more frequent KMC was associated with better weight gain in their infants.

During KMC, as I held my baby, my breasts would tingle, and milk would flow. (P9)

KMC has a positive effect on the baby's nutrition. When I did KMC, the baby's nutrition improved. His burping improved. His vomiting stopped, or he did not vomit. Thank God, the baby's weight also improved. (P4)

#### 3.3.2 Calming

Mothers participating in the study stated that KMC improved sleep quality and increased the infant's sleep duration. They also described their infants as appearing more relaxed during KMC. Mothers noticed that their infants moved less while being held skin‐to‐skin than when they were in the incubator, which they interpreted as a sign of comfort in their arms. They further reported that infants cried less during KMC. Even when the infant was crying, and the mother immediately started KMC, the infant stopped crying.

During KMC, my baby would calm down; very calm. When I put him down, he would move a lot, but not at all when he was in my arms. I could see that he was comfortable in my arms. (P10)

During KMC, the baby does not make a sound; he sleeps comfortably, no matter how long he is on my chest. (P1)

#### 3.3.3 Stability

Most mothers perceived that KMC contributed to stabilizing and improving their infants' breathing. As most preterm infants admitted to the NICU required respiratory support, the infants' need for supplemental oxygen was one of the mothers' concerns because they looked forward to the day of discharge. Mothers described with surprise that, during KMC, their infants appeared to need less supplemental oxygen, and they believed that KMC facilitated the process of weaning from oxygen support.

When doing KMC, I was paying attention to the monitor. The saturation was always 99. KMC is effective. Even the baby's position during KMC greatly affects the baby's breathing. (P3)

KMC reduced the baby's need for oxygen. As it continued, my baby was taken off the oxygen. (P2)

## 4 Discussion

The present study examined the lived experiences of mothers with preterm infants regarding KMC. The findings were summarized in three main themes: development of maternal attachment, gradual empowerment, and gaining confidence through positive outcomes.

In this study, the first theme was the development of maternal attachment. Mothers perceived KMC as the main factor in attachment between mother and infant. By performing KMC, participating mothers experienced a sense of motherhood, enjoyed interacting with their newborns, and strengthened their emotional bond with their infants. This finding was consistent with the results of some other studies. Mother‐infant attachment has been identified as the most important factor in empowering mothers to perform KMC. Mothers are not only able to understand and accept KMC but also find it enjoyable (Seidman et al. 2015). In a quasi‐experimental study, mother‐infant attachment levels were significantly higher in mothers in the KMC group than in the control group (Mehrpisheh et al. 2022). Qualitative studies also support this finding. For example, in Aziz's study, most mothers had a positive experience of performing KMC. They mentioned feelings of joy, excitement, and peace and developing a sense of motherhood to describe KMC (Aziz 2024). Another study found that performing KMC was an opportunity for mother‐infant interaction. Mothers expressed happiness with being close to their infants during KMC (Sjömar et al. 2023). KMC allows mothers to be with their infants at all times (Ndou et al. 2021), spend time with their newborns, and develop greater intimacy, making it valuable to mothers (Randa and Malesela 2023). A study in Nigeria found that most mothers felt happy when they performed KMC (Olawuyi et al. 2021). Mothers participating in a study in Brazil stated that KMC plays an important role in establishing an emotional bond between mother and infant. They described this bond as an experience they had never had before, an unconditional love or an indescribable feeling (Cantanhede et al. 2020).

The second theme of our study was gradual empowerment. Mothers became empowered during their hospitalization through their efforts, overcoming fears, worries, and challenges, and receiving motivational support from nurses and physicians regarding KMC. Other studies have had similar findings regarding mothers' fear of harming their babies. For example, a systematic review noted that mothers experienced a lot of fear and anxiety when starting KMC. Some mothers were afraid that their infants would fall or that they might damage the infants' umbilical cord when standing or moving during KMC (Bayo et al. 2022). Mothers described experiencing significant levels of fear and anxiety about the newborn and possible damage to the infant and umbilical cord as barriers to performing KMC (Aziz 2024). The mothers stated that it was difficult for them to perform KMC at first; they were afraid of lifting the infant, afraid of facing the condition of a preterm infant that requires constant care, and afraid of making mistakes and failing, which caused them considerable anxiety. Nurses assisted them in successfully performing KMC (Cantanhede et al. 2020).

In our study, the mothers attempted to perform KMC regularly and developed the necessary skills despite some issues. Similarly, a qualitative study found that mothers who performed KMC and observed positive outcomes for the infants and themselves achieved a sense of caregiver empowerment. They did not consider some minor issues as obstacles to performing KMC. They stated that holding the newborn on their chest during KMC was not difficult, and they even interpreted it as an opportunity to get closer to the infant (Sjömar et al. 2023). Similar studies have emphasized the necessity and importance of the staff educating mothers about KMC. Educating mothers about KMC and its benefits creates a positive attitude towards KMC (Chisenga et al. 2015). Mothers themselves demand education about KMC, especially its importance in promoting infant health and its impact on improving breastfeeding and creating peace of mind for both mother and infant (Aziz 2024). A study in Bangladesh reported that the participating mothers appreciated the physicians and nurses as KMC teachers, as they taught mothers how to perform KMC and encouraged and supported them in performing KMC. They also provided information about the benefits of skin‐to‐skin contact for the newborn, including weight gain, brain and lung development, and a stronger immune system (Sjömar et al. 2023). Ndou's study also showed that nurses play a crucial role in the KMC unit. They help and support mothers by providing necessary information and basic amenities (Ndou et al. 2021). A mixed‐method study reported that most mothers accepted KMC as a care method after nurses provided them with information and education through good communication. In the qualitative part of the same study, participants highlighted the importance of providing information, education, and communication in influencing mothers' acceptance of KMC (Kampekete et al. 2018). Conversely, a study in Ethiopia showed that mothers and family members did not immediately accept KMC due to a lack of awareness and information about KMC and later refused to practice KMC. The authors interpreted the reason as incorrect cultural and social beliefs about preterm infants and their growth and development (Bilal et al. 2021).

The third central theme was gaining confidence through positive outcomes. Mothers perceived positive changes in their infants' health that they attributed to KMC. This finding was consistent with the results of previous studies. In the study of Mehrpisheh et al., the mothers in the KMC group breastfed their infants more frequently than the control group. In addition, infants in the KMC group weighed significantly more at discharge (Mehrpisheh et al. 2022). Mothers participating in a qualitative study reported experiencing remarkable growth and development in their infants since they began KMC (Randa and Malesela 2023). Mothers' awareness of the benefits of KMC, especially the observation of weight gain in preterm infants, is a motivating factor for mothers to continue performing KMC (Bayo et al. 2022). The mothers said they understood the relationship between KMC and having a healthy infant. The desire to see a healthy and growing newborn motivated mothers to perform KMC (Sjömar et al. 2023). During KMC, participants could feel their infants' reactions as they became calm and relaxed. This, in turn, strengthened their sense of making a difference for their infants and of interpreting and responding to their infants' needs (Lilliesköld et al. 2022). A mixed‐method study concluded that KMC significantly improved the physiological health of preterm infants. During the study period, the infants who received KMC significantly improved various health indicators, including heart rate, respiratory rate, and blood oxygen saturation levels (Posan et al. 2025). Additionally, mothers of preterm infants in a study in Uganda experienced the effect of KMC on stabilizing the infant's body temperature and skin colour (Naloli et al. 2021).

This study highlights the important role of nurses in supporting KMC as an essential component of neonatal care. Nurses act as facilitators by educating mothers about the benefits and techniques of KMC, encouraging its regular implementation, and providing emotional and motivational support. By promoting maternal confidence and attachment, nursing interventions contribute to improved infant outcomes and maternal well‐being. The findings suggest that integrating KMC into routine neonatal care and nursing education can enhance family‐centered care and strengthen mother–infant relationships.

Although the primary aim of this interpretive phenomenological study was to explore the shared meanings of mothers' lived experiences rather than compare experiences across participant characteristics, individual factors such as maternal age, parity, and previous parenting experience may have influenced how mothers perceived and engaged in KMC. Most participants in the present study were primiparous mothers, who may have experienced greater uncertainty and emotional vulnerability because of their limited prior caregiving experience. Conversely, multiparous mothers may have drawn upon previous parenting experiences when adapting to the NICU environment. These individual differences were not the focus of the present analysis; however, they may provide additional insights into mothers' experiences and warrant further exploration in future interpretive phenomenological research.

## 5 Implications for Nursing Practice

The findings highlight the pivotal role of neonatal nurses in fostering a supportive, family‐centered environment that facilitates mothers' engagement in kangaroo mother care (KMC). Nursing practice should emphasize individualized education, hands‐on coaching, emotional reassurance, and ongoing encouragement to help mothers overcome fear and uncertainty while gradually building confidence in providing KMC. Encouraging mothers' active participation, addressing their emotional and informational needs, and delivering consistent professional support may enhance maternal empowerment, strengthen mother–infant attachment, and promote the successful integration of KMC into routine neonatal care.

## 6 Limitations

Like all studies, the present study has certain limitations. First, fathers were not included because, in the study context, mothers typically assume the primary caregiving role and are more continuously present with their newborns during hospitalization. Therefore, the findings primarily reflect mothers' experiences of providing KMC. Future research should explore fathers' and other family members' experiences of KMC, particularly in settings where fathers have greater involvement in infant care.

Second, this study was conducted in a single tertiary neonatal center with a continuous KMC ward structure, where mothers could remain with their infants for extended periods. This specific clinical and sociocultural context may have influenced mothers' experiences and perceptions of KMC and may limit the transferability of the findings to other healthcare settings with different models of KMC provision. Although the sample size was appropriate for achieving data saturation within the phenomenological design, the relatively small sample and context‐specific nature of the study should be considered when interpreting the findings.

Finally, because interviews were conducted within the clinical environment, participants' responses may have been influenced by social desirability bias. Although efforts were made to establish a comfortable, private, and trusting atmosphere that encouraged open expression, the clinical setting itself may have affected how mothers described their experiences.

## 7 Conclusion

The present study showed that performing KMC allows the mother to get closer to the infant and establish an emotional bond and attachment. Understanding this opportunity and the perceived benefits of KMC for their infants' health can, in turn, motivate mothers to practice KMC more frequently and more effectively. In addition, setting up support groups for mothers allows them to share their positive and negative experiences and support each other. Moreover, this study emphasized the importance of proper training, continuous support, adequate motivation, and close monitoring of KMC by neonatologists and nurses to support the optimal implementation of KMC. The findings of our study can be used to develop educational content for nurses and physicians for optimal implementation of KMC. Explaining the benefits of KMC for mothers and infants, identifying mothers' concerns about performing KMC and providing appropriate strategies and techniques for encouraging and strengthening the mothers' morale to overcome challenges can be considered.

## Author Contributions

**Marzieh Avazeh:** investigation, writing – original draft, data curation, resources, methodology, conceptualization, formal analysis. **Mohammadbagher Hosseini:** methodology, data curation, supervision. **Leili Borimnejad:** methodology, writing – review and editing, supervision, validation. **Mahnaz Jabraeili:** conceptualization, writing – original draft, methodology, validation, writing – review and editing, formal analysis, supervision. **Shahla Shahbazi:** conceptualization, writing – original draft, methodology, validation, writing – review and editing, formal analysis, supervision.

## Funding

This work was supported by the Research Deputy of Tabriz University of Medical Sciences.

## Disclosure

AI use statement: The authors did not use generative artificial intelligence (AI) or AI‐assisted technologies in the writing, analysis, or preparation of this manuscript.

## Ethics Statement

This study was approved by the Ethics Committee of Tabriz University of Medical Sciences (IR.TBZMED.REC.1402.655). The principles mentioned in the Declaration of Helsinki were observed in this study. Participants received adequate information about the study's purpose and methods and were ensured of their anonymity. All participants provided written informed consent before entering the study. Their participation in this study was voluntary, and they had the right to withdraw from the study at any stage. We confirm that all methods were performed in accordance with the relevant guidelines and regulations.

## Conflicts of Interest

The authors declare no conflicts of interest.

## Data Availability

The data that support the findings of this study are available from the corresponding author (M.J.) upon reasonable request.
